# Comparison of multilocus genotyping and a commercial *beta-giardin* qPCR assay for detection of *Giardia duodenalis* zoonotic assemblages in cat and dog samples

**DOI:** 10.1186/s13071-026-07342-z

**Published:** 2026-04-08

**Authors:** Andrea V. Scorza, Christian M. Leutenegger, Cecilia Lozoya, Jeffrey Tereski, Samantha Loo, Pablo D. Jimenez Castro, Michael R. Lappin

**Affiliations:** 1 Center for Companion Animal Studies, Department of Clinical Sciences, College of Veterinary Medicine and Biomedical Sciences, Colorado State University, Fort Collins, CO 80523 USA; 2 Antech Diagnostics, Inc., Mars Petcare Science & Diagnostics, Loveland, CO 80538 USA; 3 Grupo de Parasitologia Veterinaria, Universidad Nacional de Colombia, Bogota, Colombia

**Keywords:** *Giardia duodenalis*, Beta giardin, qPCR, Multilocus genotyping, Dogs, Cats

## Abstract

**Background:**

*Giardia duodenalis* is a protozoan of dogs and cats that comprises several genotypes (assemblages A–G). The common assemblages affecting small animals (C, D, F, G) are not associated with disease in humans. However, assemblages A and B can be zoonotic. Several assays can be used for detection of *G. duodenalis*, but there is a need for an accurate and faster detection method for *G. duodenalis* zoonotic assemblages. The aim of this study was to compare a *beta-giardin* quantitative polymerase chain reaction (PCR) (*bg*-qPCR) to a standard multilocus genotyping technique for detection of *G. duodenalis* zoonotic assemblages in dog and cat feces.

**Methods:**

Canine and feline fecal samples submitted to a commercial laboratory (Antech Diagnostics) that tested positive for *Giardia* by centrifugal flotation, *Giardia* enzyme-linked immunosorbent assay (ELISA), and quantitative small subunit ribosomal ribonucleic acid PCR or those positive for *Giardia* spp. by a commercial direct fluorescent assay were included in this study (140 cases with zoonotic and non-zoonotic assemblages). The *bg*-qPCR assay was optimized and then all samples were assessed by both methods. Agreement among the two methods was assessed by Cohen’s kappa agreement.

**Results:**

A total of 140 samples that were previously positive for *Giardia* were analyzed by both *bg*-qPCR and multilocus genotyping. Both *bg*-qPCR and multilocus genotyping identified 70 of 76 (92.10%) of assemblage A and B samples. A total of 64 (*n* = 40 for assemblage A and *n* = 24 for assemblage B) of 76 samples yielded concordant results, with both diagnostic techniques giving a Cohen’s kappa agreement value of 0.828. Non-zoonotic assemblages were amplified only by multilocus genotyping in 55 samples, and 9 samples were negative for both methods. Multilocus genotyping reported mixed infections in 23 cases and included A/D (1 dog), B/C (1 dog), B/F (1 dog; 2 cats), and C/D (18 dogs).

**Conclusions:**

For the zoonotic assemblages, the results between multilocus genotyping and the *bg*-qPCR agreed for most cases. These data support the *bg*-qPCR as a less expensive and laborious option to determine zoonotic *Giardia* A/B assemblages in feces of dogs and cats.

**Graphical Abstract:**

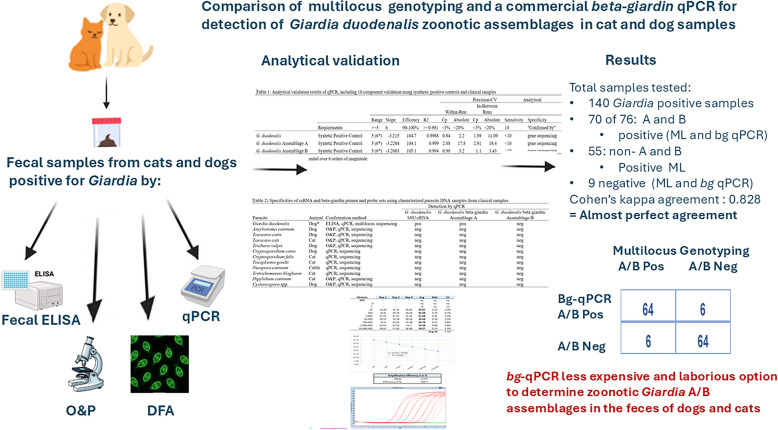

## Background

*Giardia duodenalis* (syn. *Giardia lamblia, Giardia intestinalis*) is a common protozoan in dogs and cats that also infects other mammals including humans. It comprises several genotypes (assemblages A–G). The most common assemblages affecting dogs and cats (C, D, F) are not associated with disease in humans. However, the zoonotic assemblages A and B can be detected in feces of some dogs and cats. While several assays have been developed to identify zoonotic assemblages of *G. duodenalis* in humans, there remains a significant need for more precise, affordable, efficient diagnostic methods with quick turnaround specifically designed to detect zoonotic assemblages of *G. duodenalis* in companion animals [1, 2]. Developing such methods is crucial to better understand the role of companion animals in zoonotic transmission and to implement effective public health measures for disease prevention.

A meta-analysis reported an overall prevalence of *Giardia* in dogs and cats of 15% and 12%, respectively [3]. However, in dogs, infection rates ranged between 1.9% and 57.9% [4]. The occurrence of *G. duodenalis* in cats varied between 1.3% and 27.3% [4]. The majority of the *G. duodenalis* isolates genotyped from dogs were dog-specific assemblages C and D [4–7]. In cats, most isolates that were genotyped were assemblage F followed by assemblage A [8].

In humans, *G. duodenalis* is also a very common protozoan and is estimated to cause approximately 280 million human cases of diarrhea per year [9]. *Giardia duodenalis* can cause a variety of clinical signs in people and companion animals, ranging from the subclinical passage of cysts in feces in some individuals to severe, acute, and potentially chronic diarrhea in others. The risk for people of acquiring *Giardia* infections directly from dogs and cats is relatively low; however, pets shedding potentially zoonotic assemblages contribute to environmental contamination. In addition, people with compromised immune systems could experience symptoms of longer duration or severe complications after contact with pathogens, including those carried by animals [10].

The molecular characterization of *Giardia duodenalis* has been conducted using several genetic markers, including* small subunit ribosomal RNA* (*SSU rRNA*), *beta-giardin* (*bg*), *glutamate dehydrogenase* (*gdh*), and *triose phosphate isomerase *(*tpi*) [11–14]. However, many primers designed to amplify the *SSU rRNA gene* target a short and conserved fragment, which limits the ability to differentiate between *G. duodenalis* assemblages [15]. As a result, protein-coding genes such as *gdh*, *bg*, and *tpi* are more commonly used for genotyping [15].

Genotyping inconsistencies have often been reported in *G. duodenalis* isolates from dogs. The choice of genetic markers can influence whether isolates are identified as host-adapted, non-zoonotic assemblages (C and D) or as zoonotic assemblages (A or B), which have the potential to infect humans [12, 16].

Owing to the variability in amplification rates of different genes and the preferential amplification of assemblages by specific primers, a multilocus genotyping approach is recommended [17]. Multilocus genotyping reduces the risk of misclassification that can occur with single-locus genotyping, provides enhanced resolution, enables the detection of mixed infections, and offers a more comprehensive understanding of host-specific and zoonotic assemblages.

Despite its advantages, multilocus genotyping has limitations. These include the potential for false-negative results due to differences in gene amplification rates and its time-intensive and costly nature [18]. While multilocus genotyping is widely considered the most informative method for characterizing *G. duodenalis* assemblages, single-locus genotyping remains a valid alternative, particularly when targeting genes with high sequence heterogeneity, such as *bg*.

*Beta-giardin* is part of a group of structural proteins that are considered unique to *Giardia* and so is a highly specific marker [19]. Several studies reported the *bg* gene to be more informative than the other genes used in multilocus analysis [20–22]. Therefore, we decided to base the quantitative polymerase chain reaction (qPCR) assay described here on the *bg* gene (*bg*-qPCR assay). The aims of this study were to optimize the performance of the *bg*-qPCR assay for detection of *G. duodenalis* zoonotic assemblages A and B in dog and cat feces and to compare the results with the standard multilocus genotyping technique using results from three genes.

## Methods

### Sample collection

Canine and feline fecal samples submitted to a commercial reference laboratory (Antech Diagnostics) that tested positive/detected for *Giardia* by at least one of the following methods: centrifugal flotation (ova and parasite [O&P]), *Giardia* enzyme-linked immunosorbent assay (ELISA), and quantitative small subunit ribosomal ribonucleic acid PCR (SSU rRNA-qPCR) were included in this study. The *SSU rRNA*-qPCR used primers previously described [11]. A subset of canine and feline samples that tested positive for *Giardia* spp. by a commercial direct fluorescent assay (Merifluor^®^ *Cryptosporidium*/*Giardia*, Meridian Bioscience Cincinnati, Ohio) was also included.

### Analytical validation of the *bg*-qPCR assay

The final primer set and methods of the *bg*-qPCR assay are proprietary and offered commercially (KeyScreen^™^ GI Parasite PCR, Antech Diagnostics, Inc.). The validation experiments described here were performed using previously established analytical parameters [23].

### DNA extraction

Feces (150 mg) and 750 µl guanidinium thiocyanate-based lysis solution were added into a bashing bead tube containing zirconia beads (Spex Sample Prep, Metuchen, NJ, USA) and then homogenized in a Mini G (Spex SamplePrep) for 7.5 min according to the manufacturer’s recommendations. Lysates were cleared by centrifugation, and 200 µl of the supernatant was used to extract total nucleic acid in a magnetic bead-based automated extractor (KingFisher Apex, Thermo Fisher, Waltham, MA, USA). Nucleic acids were eluted in 120 µl of nuclease-free water and 5 µl used in 12 µl total volume real-time PCR reactions.

### PCR design

*Giardia duodenalis* assemblage A- and B-specific hydrolysis probes were adapted from previously published regions within the *bg* gene targeting a variable region around nucleotide position 473 (KM190684 [24];). Hydrolysis probes included locked nucleic acids (IDT, Coralville) [25]. Assemblage specific multisequence alignments were used to design additional primers than previously published allowing amplification of more recently deposited sequences across the different *G. duodenalis* assemblages [26]. Flanking sequencing primers amplifying a 335-base pair (bp) region of the coding sequence of the *bg* gene allowed amplification and sequence confirmation of a larger fragment of the *Giardia bg* amplicon described in this study. The sequence data from *G. duodenalis* isolates by the *bg*-qPCR was compared by Basic Local Alignment Search Tool (BLAST) analysis with sequences from the nucleotide database from the GenBank (National Center for Biotechnology Information (NCBI) BLAST, National Center for Biotechnology Information, Bethesda, MD, USA).

Analytical validation was determined using synthetic positive controls designed by overlapping the real-time PCR primers by 15 nucleotides (Table 1). All qPCR reactions were run in an LC480 (Roche, Indianapolis, IN, USA) using standard cycling parameters according to the manufacturer. qPCR reactions contained KAPA3G master mix in combination with uracil–DNA–glycosylate (UNG) to prevent PCR product carry-over (Roche, Indianapolis, IN, USA) in a total volume of 12 µl.

**Table 1:** Analytical validation results of qPCR, including ten-component validation using synthetic positive controls and clinical samples

								Precision CV		Analytical	
						Within-run		In-between runs			
		Range	Slope	Efficiency	*R*^2^	Cp	Absolute	Cp	Absolute	Sensitivity	Specificity
	Requirements	> = 5	b	90–110%	> = 0.991	< 3%	< 20%	< 3%	< 20%	10	“Confirmed by”
*G. duodenalis*	Synthetic positive control	5 (6*)	−3.215	104.7	0.9988	0.84	2.2	1.89	11.09	< 10	Gene sequencing
*G. duodenalis* assemblage A	Synthetic positive control	5 (6*)	−3.2264	104.1	0.999	2.88	17.8	2.91	18.4	< 10	Gene sequencing
*G. duodenalis* assemblage B	Synthetic positive control	5 (6*)	−3.2063	105.1	0.994	0.98	3.2	1.1	3.43	< 10	Gene sequencing

6*, actual standard curve extended over six orders of magnitude

### Analytical sensitivity

Synthetic nucleic acids from assemblage A and B were synthesized using solid-phase phosphoramidite chemistry (Ultramer, IDT DNA, Coralville, Iowa) and used as positive controls. Concentrations were determined by the manufacturer. These were tenfold serially diluted to determine the limit of detection (LOD). Each dilution was run in triplicate to calculate averages, standard deviations, and coefficients of variation (CVs). In addition, fecal samples that tested positive for *G. duodenalis* O&P and SSU rRNA-qPCR were used to quantify cysts using a commercial direct immunofluorescence assay. Two samples with high concentrations of *Giardia* cysts (40,000 and 50,000 cysts per gram, respectively) were serially diluted to achieve final concentrations of 4 and 5 cysts per gram. For each sample, three slides were prepared, and *Giardia* cysts were counted using a fluorescent microscope. Nucleic acids extracted from these preparations were then serially diluted in tenfold increments to determine the limit of detection (LOD) of the assay.

### Analytical specificity

Nucleic acids were extracted from samples that tested positive for *Cystoisospora* spp., *Ancylostoma* spp., *Cryptosporidium canis*, *Cryptosporidium felis*, *Dipylidium caninum*, *Toxoplasma gondii*, *Neospora caninum*, *Tritrichomonas blagburni*, *Toxocara canis*, *Toxocara cati*, and *Trichuris vulpis* at either fecal zinc sulfate-based centrifugation and flotation, ELISA, or qPCR for each of the parasites (Table 2). These qPCR tests were confirmed for analytical specificity by using outside sequencing primers with conventional Sanger sequencing and BLAST analysis (NCBI BLAST, National Center for Biotechnology Information, Bethesda, MD, USA) as previously described [30]. The extracted nucleic acid was then assessed using *Giardia bg* qPCR.

**Table 2:** Specificities of *SSU rRNA* and *beta-giardin* primer and probe sets using characterized parasite DNA samples from clinical samples

			Detection by qPCR		
			*G. duodenalis*	*G. duodenalis beta giardin*	*G. duodenalis beta giardin*
Parasite	Animal	Confirmation method	*SSU rRNA*	Assemblage A	Assemblage B
*Giardia duodenalis*	Dog^*^	ELISA, qPCR, multilocus sequencing	Positive	Positive	Negative
*Ancylostoma caninum*	Dog	O&P, qPCR, sequencing	Negative	Negative	Negative
*Toxocara canis*	Dog	O&P, qPCR, sequencing	Negative	Negative	Negative
*Toxocara cati*	Cat	O&P, qPCR, sequencing	Negative	Negative	Negative
*Trichuris vulpis*	Dog	O&P, qPCR, sequencing	Negative	Negative	Negative
*Cryptosporidium canis*	Dog	qPCR, sequencing	Negative	Negative	Negative
*Cryptosporidium felis*	Cat	qPCR, sequencing	Negative	Negative	Negative
*Toxoplasma gondii*	Cat	qPCR, sequencing	Negative	Negative	Negative
*Neospora caninum*	Cattle	qPCR, sequencing	Negative	Negative	Negative
*Tritrichomonas blagburni*	Cat	qPCR, sequencing	Negative	Negative	Negative
*Dipylidium caninum*	Cat	O&P, qPCR, sequencing	Negative	Negative	Negative
*Cystoisospora* spp.	Dog	O&P, qPCR, sequencing	Negative	Negative	Negative

^*^Sample no. 4z from Table 3

### Multilocus genotyping method

The *G. duodenalis*-nested PCR assays and the sequencing of the amplified genes *gdh*, *bg*, *tpi* generic, and *tpi* dog-specific were performed using published protocols with some modifications [6, 12–14, 27]. In the *gdh* protocol, 24 µl per reaction of a commercial master mix (Hot Start Qiagen Master Mix, Qiagen, Germantown, Maryland, USA) and 24 µl of water were used as a replacement for the master mix described in the original publication. Furthermore, 2 µl of genomic DNA were included per reaction, instead of 1 µl, as specified in the original protocol [12].

For the *bg* protocol, 24 µl per reaction of the same commercial master mix (Hot Start Qiagen Master Mix, Qiagen, Germantown, Maryland, USA) and 24 µl of water were used, replacing the master mix described in the original publication [14].

In the *tpi* protocol, the annealing temperatures for the first and second amplification reactions were adjusted to 51 °C and 55 °C, respectively, compared with the 50 °C annealing temperature described in the original protocol [13]. In addition, the total reaction volume was reduced to 50 µl, whereas the original protocol specified a volume of 100 µl [13]. The thermal protocols of the *gdh* and *bg* assays were identical to those described in the original methods.

DNA sequences were analyzed in forward direction using an ABI3100 Genetic Analyzer (Applied Biosystems, Foster City, California). The sequence data from *G. duodenalis* isolates by the three genes was compared by BLAST analysis with sequences from the nucleotide database from the GenBank (NCBI BLASTn, National Center for Biotechnology Information, Bethesda, MD, USA). Sequences were also aligned with reference strains of assemblage A sub-assemblages to confirm sub-assemblage identity [4]. Representatives of the nucleotide sequences generated in this study were placed in GenBank under the accession numbers OQ434171–OQ434188.

### Methods of analysis/assay comparisons

Cohen’s kappa was used to determine the level of agreement between the *bg*-qPCR and multilocus genotyping (MLG) results for assemblage A and B. Values range from 0.01 to 0.20: none to slight agreement to 0.21–0.40: fair agreement, 0.41–0.60: moderate agreement, 0.61–0.80: substantial agreement, and 0.81–1.00: almost perfect agreement. For the calculation of Cohen’s kappa, samples were classified in five groups (sort key presented in Table 3):

- 1 (Sort key 1): A/B *bg*-qPCR positive/A/B MLG positive;
- 2 (Sort key 2): A/B *bg*-qPCR positive/A/B MLG negative or assemblages CDFG;
- 3 (Sort key 3): A/B *bg*-qPCR negative/A/B MLG positive;
- 4 (Sort key 4): A/B *bg*-qPCR negative/assemblages CDF MLG positive, and
- 5 (Sort key 5): A/B *bg*-qPCR negative/A/B MLG negative.

**Table 3:** Comparison of *bg*-qPCR and multilocus genotyping results

	Sample ID	Species	*bg*-qPCR	Multilocus genotyping genes					*Original classification*	*Sort key*
				*BG*	*GDH*	*TPI*—generic primers	*TPI*—dog-specific primers	Assemblage classification multilocus		
	1z	Canine	A	AI	A	A	Negative	A	A	1
	2z	Canine	A	AI	A	A	Negative	A	A	1
	4z	Canine	A	A	A	A	Negative	A	A	1
	5z	Canine	B	B	B	C	Negative	B/C	B	1
	6z	Canine	B	B	B	B	Negative	B	B	1
	9z	Canine	B	B	BIII	BIII	Negative	B	B	1
	10z	Canine	B	B	BIII	B	B	B	B	1
	11z	Canine	B	B	B	BIII	BIII	B	B	1
	12z	Canine	B	F	B	BIII	Negative	F/B	B	1
	13z	Canine	B	B	BIV	BIII	Negative	B	B	1
	8122	Canine	A	AI	A	A	AI	A	A	1
	8089	Canine	A	A	Negative	Negative	Negative	A	A	1
	CSU 5	Canine	A	A	A	A	Negative	A	A	1
	CSU 7	Canine	A	Negative	Negative	A	Negative	A	A	1
	CSU 8	Canine	B	B	B	B	B	B	B	1
	CSU 11	Canine	A	A	Negative	AI	Negative	A	A	1
	CSU 12	Canine	B	Negative	BIV	B	B	B	B	1
	CSU 13	Canine	A	A	A	A	Negative	A	A	1
	CSU 17	Canine	A	A	A	AI	A	A	A	1
	CSU 19	Canine	A	A	A	AI	AI	A	A	1
	CSU 29	Canine	B	BIII	BIV	B	Negative	B	B	1
	CSU 30	Canine	B	BIV	Negative	B	Negative	B	B	1
	CSU 35	Canine	A	A	A	AI	Negative	A	A	1
	CSU 39	Canine	A	A	A	AI	AI	A	A	1
	CSU 40	Canine	B	B	BIV	B	B	B	B	1
	CSU 44	Canine	A	Negative	A	A	A	A	A	1
	CSU 45	Canine	A	A	A	AI	Negative	A	A	1
	CSU 49	Canine	A	Negative	A	AI	AI	A	A	1
	8z	Canine	B	G	G	Negative	Negative	G	G	2
	CSU 26	Canine	A	Negative	Negative	Negative	Negative	Negative	Negative	2
	CSU 27	Canine	A	Negative	Negative	Negative	Negative	Negative	Negative	2
	CSU 2	Canine	A/B undetected	A	D	Negative	D	A/D	A/D	3
	CSU 6	Canine	A/B undetected	Negative	A	A	Negative	A	A	3
	CSU 10	Canine	A/B undetected	BIV	BIV	B	Negative	B	B	3
	CSU 64	Canine	A/B undetected	BIII	B	Negative	Negative	B	B	3
	14nz	Canine	A/B undetected	C	C	Negative	C	C	Negative	4
	15nz	Canine	A/B undetected	C	C	C	C	C	Negative	4
	16nz	Canine	A/B undetected	C	C	C	C	C	Negative	4
	17nz	Canine	A/B undetected	C	C	Negative	C	C	Negative	4
	18nz	Canine	A/B undetected	D	D	Negative	D	D	Negative	4
	19nz	Canine	A/B undetected	C	C	C	C	C	Negative	4
	22nz	Canine	A/B undetected	D	D	Negative	D	D	Negative	4
	23nz	Canine	A/B undetected	D	D	Negative	D	D	Negative	4
	24nz	Canine	A/B undetected	D	D	C	D	D/C	Negative	4
	25nz	Canine	A/B undetected	D	D	Negative	D	D	Negative	4
	26nz	Canine	A/B undetected	D	D	Negative	D	D	Negative	4
	27nz	Canine	A/B undetected	D	D	Negative	D	D	Negative	4
	28nz	Canine	A/B undetected	D	D	Negative	D	D	Negative	4
	29nz	Canine	A/B undetected	C	C	Negative	C	C	Negative	4
	30nz	Canine	A/B undetected	C	C	C	D	C/D	Negative	4
	31nz	Canine	A/B undetected	D	Negative	Negative	D	D	Negative	4
	32nz	Canine	A/B undetected	D	D	Negative	D	D	Negative	4
	33nz	Canine	A/B undetected	D	D	Negative	D	D	Negative	4
	34nz	Canine	A/B undetected	D	D	Negative	D	D	Negative	4
	35nz	Canine	A/B undetected	D	D	Negative	D	D	Negative	4
	36nz	Canine	A/B undetected	C	C	C	C	C	Negative	4
	6113	Canine	A/B undetected	C	C	Negative	Negative	C	Negative	4
	6114	Canine	A/B undetected	C	C	Negative	C	C	Negative	4
	6143	Canine	A/B undetected	C	C	Negative	D	C/D	Negative	4
	6144	Canine	A/B undetected	C	C	Negative	D	C/D	Negative	4
	6137	Canine	A/B undetected	C	C	Negative	D	C/D	Negative	4
	6145	Canine	A/B undetected	D	D	Negative	C	D/C	Negative	4
	6146	Canine	A/B undetected	D	D	Negative	D	D	Negative	4
	6147	Canine	A/B undetected	D	D	Negative	C	D/C	Negative	4
	6140	Canine	A/B undetected	C	C	Negative	C	C	Negative	4
	6268	Canine	A/B undetected	D	Negative	Negative	Negative	D	Negative	4
	6269	Canine	A/B undetected	D	D	Negative	C	D/C	Negative	4
	6282	Canine	A/B undetected	D	D	Negative	Negative	D	Negative	4
	6284	Canine	A/B undetected	D	Negative	Negative	C	C/D	Negative	4
	6285	Canine	A/B undetected	D	D	Negative	C	D/C	Negative	4
	6286	Canine	A/B undetected	C	Negative	Negative	C	C	Negative	4
	6786	Canine	A/B undetected	C	D	Negative	D	C/D	Negative	4
	6782	Canine	A/B undetected	C	C	Negative	D	C/D	Negative	4
	6783	Canine	A/B undetected	C	Negative	Negative	D	C/D	Negative	4
	6785	Canine	A/B undetected	D	C	Negative	C	D/C	Negative	4
	6901	Canine	A/B undetected	C	C	Negative	D	C/D	Negative	4
	6905	Canine	A/B undetected	D	Negative	Negative	Negative	D	Negative	4
	CSU 62	Canine	A/B undetected	F	Negative	Negative	Negative	F	Negative	4
	CSU 63	Canine	A/B undetected	F	Negative	Negative	Negative	F	Negative	4
	8095	Canine	A/B undetected	D	D	Negative	C	D/C	Negative	4
	8125	Canine	A/B undetected	C	C	C	D	C/D	Negative	4
	6149	Canine	A/B undetected	Negative	Negative	Negative	Negative	Negative	Negative	5
	6134	Canine	A/B undetected	Negative	Negative	Negative	Negative	Negative	Negative	5
	6912	Canine	A/B undetected	Negative	Negative	Negative	Negative	Negative	Negative	5
	6897	Canine	A/B undetected	Negative	Negative	Negative	Negative	Negative	Negative	5
	6898	Canine	A/B undetected	Negative	Negative	Negative	Negative	Negative	Negative	5
	6906	Canine	A/B undetected	Negative	Negative	Negative	Negative	Negative	Negative	5
	6907	Canine	A/B undetected	Negative	Negative	Negative	Negative	Negative	Negative	5
	7998A	Canine	A/B undetected	Negative	Negative	Negative	Negative	Negative	Negative	5
	8106	Canine	A/B undetected	Negative	Negative	Negative	Negative	Negative	Negative	5
	3z	Feline	A	A	AI	AI	Negative	A	A	1
	7z	Feline	B	B	B	BIII	Negative	B	B	1
	7125	Feline	A	A	A	A	Negative	A	A	1
	7126	Feline	A	A	A	A	Negative	A	A	1
	7146	Feline	A	A	A	A	Negative	A	A	1
	7168	Feline	B	B	BIV	BIII, BIV	Negative	B	B	1
	7203	Feline	B	B	BIV	B	Negative	B	B	1
	CSU 1	Feline	A	A	A	Negative	Negative	A	A	1
	CSU 3	Feline	A	A	AI	AI	Negative	A	A	1
	CSU 4	Feline	A	AI	A	AI	AI	A	A	1
	CSU 9	Feline	A	A	A	AI	Negative	A	A	1
	CSU 14	Feline	A	A	A	A	A	A	A	1
	CSU 15	Feline	B	F	BIV	F	BIII	B	B	1
	CSU 16	Feline	A	A	A	A	AI	A	A	1
	CSU 18	Feline	B	BIII	BIV	B	Negative	B	B	1
	CSU 20	Feline	A	A	AI	AI	AI	A	A	1
	CSU 21	Feline	A	A	A	AI	AI	A	A	1
	CSU 22	Feline	B	BIII	BIV	B	Negative	B	B	1
	CSU 23	Feline	A	AI	A	A	AI	A	A	1
	CSU 24	Feline	B	BIII	BIV	B	Negative	B	B	1
	CSU 25	Feline	B	BIII	Negative	B	Negative	B	B	1
	CSU 28	Feline	A	A	A	Negative	Negative	A	A	1
	CSU 31	Feline	A	A	Negative	AI	AI	A	A	1
	CSU 32	Feline	B	BIII	BIV	B	Negative	B	B	1
	CSU 33	Feline	A	A	Negative	Negative	Negative	A	A	1
	CSU 34	Feline	A	A	A	AI	AI	A	A	1
	CSU 36	Feline	A	AI	A	AI	AI	A	A	1
	CSU 37	Feline	A	A	AI	AI	Negative	A	A	1
	CSU 38	Feline	A	A	A	AI	Negative	A	A	1
	CSU 41	Feline	B	BIII	BIV	B	B	B	B	1
	CSU 42	Feline	B	BIII	BIV	B	B	B	B	1
	CSU 43	Feline	A	A	A	AI	AI	A	A	1
	CSU 46	Feline	A	AI	A	A	Negative	A	A	1
	CSU 47	Feline	A	Negative	A	AI	AI	A	A	1
	CSU 48	Feline	A	Negative	A	AI	AI	A	A	1
	CSU 50	Feline	B	B	BIV	B	B	B	B	1
	7097	Feline	B	Negative	Negative	Negative	Negative	Negative	Negative	2
	7166	Feline	B	Negative	Negative	Negative	Negative	Negative	Negative	2
	7182	Feline	A	Negative	Negative	Negative	Negative	Negative	Negative	2
	37nz	Feline	A/B undetected	F	F	BIII	Negative	F/B	B	3
	CSU 52	Feline	A/B undetected	B	BIV	B	Negative	B	B	3
	20nz	Feline	A/B undetected	F	F	Negative	Negative	F	Negative	4
	21nz	Feline	A/B undetected	F	F	Negative	Negative	F	Negative	4
	8130	Feline	A/B undetected	D	D	Negative	C	D/C	Negative	4
	8131	Feline	A/B undetected	D	D	Negative	D	D	Negative	4
	8132	Feline	A/B undetected	D	Negative	Negative	Negative	D	Negative	4
	8133	Feline	A/B undetected	D	D	Negative	D	D	Negative	4
	8134	Feline	A/B undetected	D	D	Negative	Negative	D	Negative	4
	CSU 51	Feline	A/B undetected	F	F	F	Negative	F	Negative	4
	CSU 55	Feline	A/B undetected	F	Negative	Negative	Negative	F	Negative	4

Reference: original classification: *SSU* rRNA-qPCR result. Sort key 1: A/B *bg*-qPCR positive/A/B MLG positive; 2: A/B *bg*- qPCR positive/A/B MLG negative or assemblages CDFG; 3: A/B *bg*-qPCR negative/A/B MLG positive; 4: A/B *bg*-qPCR negative/assemblages CDF MLG, and 5: A/B *bg*-qPCR negative/A/B MLG negative

In addition, diagnostic sensitivity, diagnostic specificity, positive predictive value (PPV), and negative predictive value (NPV) of the *bg-*qPCR assay in comparison with the MLG were calculated.

In the MLG, the assemblage of each sample was determined on the basis of the genotyping results of one or more genes. When results were not in agreement, all the assemblages detected by the different genes were assigned to that sample and were considered mixed infections.

## Results

### Sample collection

A total of 140 fecal samples from dogs (*n* = 90, 64.29%) and cats (*n* = 50, 35.71%) that tested positive/detected for *Giardia* spp. by at least one of the following methods: centrifugal flotation (ova and parasite, O&P), *Giardia* ELISA (TechLab Giardia II, Thermo Fisher Scientific, Waltham, Massachusetts), and quantitative small subunit ribosomal ribonucleic acid PCR (*SSU rRNA-qPCR*) were included in this study [11]. The sampling distribution was: Antech Diagnostics (*n* = 84) and the Center for Companion Animal Studies (*n* = 56).

### Analytical validation of the *bg*-qPCR assay

**Fig. 1:**
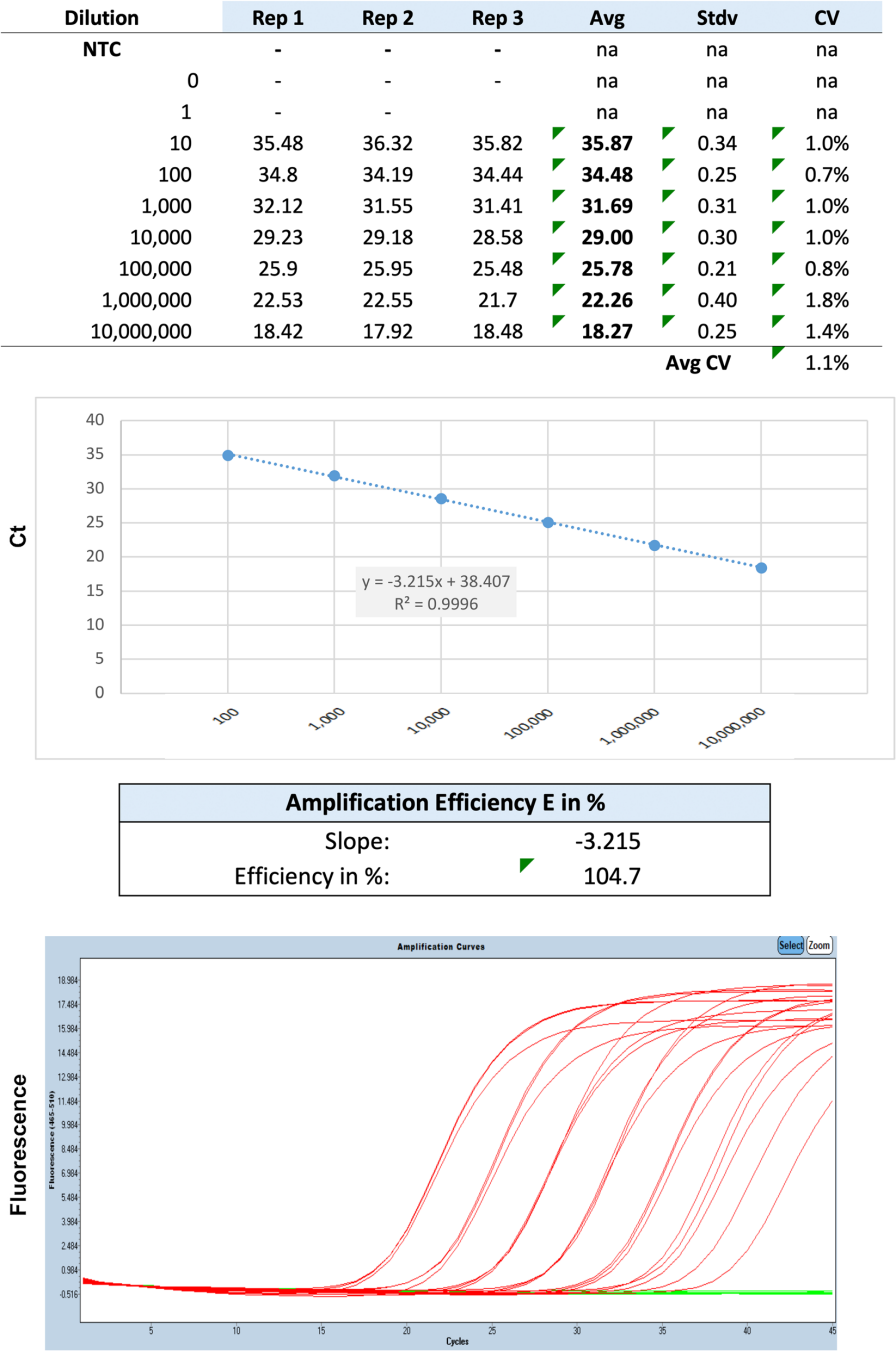
Detection of analytical sensitivity for the *bg*-qPCR

Analytical sensitivity: Amplification of serial dilutions showed that this assay detected 10 copies per 5 µl of synthetic fragment DNA reproducibility (Table 1). The standard curve displayed good linearity between 1 × 10^1^ and 1 × 10^7^ copies per 5 µl. This *bg*-qPCR assay was performed with an efficiency of 104.7% and *R*^2^ values were greater than 0.99 (Fig. 1). This *bg*-qPCR had good repeatability with intra- and inter-assay coefficients of variation less than 1.1%. When using enumerated cysts to obtain limit of detection information, the lowest detection limit obtained was 8.9 cysts per gram of feces.

Analytical specificity: The *bg*-qPCR assay amplified DNA from *Giardia* but not the other parasites tested (Table 2).

### Multilocus genotyping results

The multilocus genotyping method detected 126 of 140 positive samples, for a detection rate of 90% (Table 3). Of the 126 positive samples, assemblages A or B were amplified from 70 samples and 56 corresponded to non-A/B assemblages. The primer set with the highest number of positive results was the *bg* gene followed by the gdh gene. Both *tpi*-generic and *tpi* dog-specific primers showed the lowest number of positive results. Among the 41 assemblage A isolates, sub-assemblage AI was identified in 7, 4, and 21 isolates on the basis of the *bg*, *gdh*, and *tpi* genes, respectively. Molecular typing of assemblage B using BLAST analysis identified two sub-assemblages, BIII and BIV. Of the 25 assemblage B isolates, sub-assemblage BIII was identified in 9, 2, and 6 isolates on the basis of the *bg*, *gdh*, and *tpi* genes, respectively. Sub-assemblage BIV was identified in 2 and 6 isolates on the basis of the *bg* and *gdh* genes.

### Method of analysis/assays comparison

When the results of multilocus genotyping and the *bg*-qPCR were compared for detection of zoonotic assemblages, the results showed a Cohen’s kappa value of 0.828. When results of the *bg*-qPCR assay were compared with those of the MLG for detection of zoonotic assemblages, the diagnostic sensitivity, specificity, positive predictive value, and negative predictive value were all 91.47%.

Non-zoonotic assemblages were amplified only by MLG in 55 samples (Table 3). Multilocus genotyping reported mixed infections in 23 cases and included A/D (1 dog), B/C (1 dog), B/F (1 dog; 2 cats), and C/D (18 dogs) (Table 3). The discrepancies between *bg*-qPCR and MLG are displayed in Table 4.

**Table 4:** Discordance between *bg*-qPCR and multilocus genotyping results

	Multilocus genotyping genes						
Sample ID	Species	*bg*-qPCR	*BG*	*GDH*	*TPI*—generic primers	*TPI*—dog specific primers	Assemblage classification multilocus
7166	Feline	B	Negative	Negative	Negative	Negative	Negative
7182	Feline	A	Negative	Negative	Negative	Negative	Negative
CSU 2	Canine	Negative	AI	D	Negative	D	Mixed A, D
CSU 6	Canine	Negative	Negative	AI	A	Negative	A
CSU 10	Canine	Negative	BIV, B	BIV	B	Negative	B
CSU 26	Canine	A	Negative	Negative	Negative	Negative	Negative
CSU 27	Canine	A	Negative	Negative	Negative	Negative	Negative
37nz	Feline	Negative	F	F	BIII	Negative	Mixed B, F
CSU 64	Canine	Negative	B, BIII	B	Negative	Negative	B
CSU 52	Feline	Negative	B	BIV	B	Negative	B

## Discussion

The aim of this study was to validate a *bg*-qPCR for detection of *G. duodenalis* assemblages with zoonotic potential A and B in dog and cat fecal samples. The *bg*-qPCR assay described in this manuscript was demonstrated to be a highly accurate assay in identifying *G. duodenalis* assemblages A and B. The analytical validation confirmed that the assemblage A and B *bg*-qPCR assay amplified with high efficiency (104.1% and 105.1%, respectively) over at least a five-log standard curve, providing a wide dynamic range to quantify *Giardia* parasite burden in clinical samples (Fig. 1).

*Beta giardin*, *gdh*, and *tpi* genes are commonly used for genotyping to the assemblage- and sub-assemblage-level *G. duodenalis* [4]. All these assays are reported to be highly specific at detecting *G. duodenalis* assemblages [4]. We chose the *bg* as the target gene for the single-gene *bg*-qPCR since giardin genes are unique to *Giardia* protozoa. Giardins are structural proteins located at the ventral disk of the trophozoites [28]. The *bg* gene includes conserved regions within assemblages with variable domains in between assemblages; these regions have been targeted using locked-nucleic acid hydrolysis probes to have the highest possible specificity for assemblages A and B [25].

The specificities of the primer and probe sets were examined by performing *bg*-qPCR assays with a panel of DNA samples from different gastrointestinal parasites of dogs and cats (Table 2). Our results agree with those of other researchers in which no cross-amplification of various gastrointestinal parasites was detected [29]. These samples were confirmed either by gene sequencing using flanking primers, by using established *SSU rRNA*-qPCR [30], and by multilocus sequencing analysis.

Regarding the analytical sensitivity of the *bg*-qPCR, the standard curves confirmed a reproducible analytical sensitivity of five molecules per PCR reaction for assemblage A and B (Fig. 1, Table 1). The detection limit of the *bg*-qPCR was 8.9 cysts per gram of feces. The detection limit of a previous *bg*-qPCR assay was performed by spiking *Giardia* DNA into *Giardia*-negative samples [26]. In the study mentioned, *Giardia*-negative PCR samples were spiked with *Giardia* DNA with dilutions that ranged from 1 to 1000 *Giardia* cysts, and DNA was amplified at every concentration [26]. We believe, however, that spiking fecal samples with *Giardia* cysts provides a more accurate representation of the protozoal burden in clinical diagnostic specimens.

Furthermore, shedding dynamics of *Giardia* cysts by cats may fluctuate from undetectable to concentrations greater than 1,000,000 cysts per gram of feces [31, 32]. Similarly, shedding *Giardia* cysts in dogs may also vary; young dogs shed an average of 2000 cysts per gram of feces, and in one study, the mean cyst count per gram of feces for all infected dogs was 705.8 [33]. The reported range in another study was between 26 and 114,486 cysts per gram of feces [34]. Therefore, we are confident that the detection limit of the *bg*-qPCR presented in this study is adequate to detect the low numbers of *Giardia* cysts shed by cats and dogs.

Both *bg*-qPCR and multilocus genotyping identified 70 of 76 (92.10%) of the A and B assemblages. The Cohen’s kappa value was 0.828, which indicates almost perfect agreement between the two methods (Table 4). The high Cohen’s kappa value and the similar detection rates demonstrate that the *bg*-qPCR method is a reliable alternative to the multilocus genotyping for identifying *G. duodenalis* potentially zoonotic assemblages A and B. The fact that the assay is simpler, less expensive, and more rapid are additional benefits.

A meta-analysis reported the prevalence of *Giardia* spp. infections in cats and dogs to be 12% and 15%, respectively [3]. Dogs and cats with giardiasis can occur without clinical signs or have acute or chronic diarrhea [3]. In general, the transmission of dog-specific assemblages is predominant, particularly when dogs are in close contact such as crowded kennels [15]. In contrast, in household dogs, with minimal interaction with other dogs, the frequency of dog-to-dog transmission could be lower and infections with potentially zoonotic assemblages in dogs could be more frequent [15].

In this study, dogs carried more D (*n* = 16) than C (*n* = 11) *Giardia* assemblages, when counting concordant assemblage identification using the multilocus genotyping PCR tests. Dogs also carried slightly more A (*n* = 20) than B (*n* = 15) assemblages. Two dogs carried F assemblages, most likely explained by coprophagic behavior. In cats, more A (*n* = 24) than B (*n *= 14) assemblages were detected. In addition, assemblage F was detected in four cats and four were identified as assemblage D. Assemblage D has occasionally been detected in cats using molecular surveys in cats and is considered a spill-over phenomenon in mixed environments [35].

Dog-specific assemblages C and D are predominant in dogs; however, assemblages A and B have been commonly detected in cats and dogs [4, 35]. Assemblage A was the dominant assemblage in dogs in studies from USA, China, Japan, Germany, Brazil, in dogs from indigenous communities Canada, and Jamaica [36–43].

Multilocus characterization of assemblage A identified three major sub-assemblages (AI, AII, and AIII) with distinct subtypes at individual loci [44]. In cats and dogs, sub-assemblage AI, considered to be host specific to animals, was the predominant subtype but was also reported in humans [15, 40, 45–49]. While sub-assemblage AII is primarily associated with human infections, it has also been detected in dogs and cats [50–53]. Similarly, sub-assemblage AIII, typically linked to wild ruminants, has also been reported in cats and dogs [54].

Our study identified 40 isolates as assemblage A using the BLASTn algorithm (Table 3). However, subtyping resolution varied significantly. While some samples were successfully classified at the sub-assemblage level, others could only be confirmed at the broader assemblage level. This limitation often arose because many samples exhibited an identical percentage of homology to reference sequences from more than one sub-assemblage simultaneously, including sub-assemblage AII that is predominant in humans.

While BLASTn is an excellent tool for initial sequence screening, the high genetic diversity and significant allelic sequence heterozygosity characteristic of *Giardia* make definitive sub-assemblage assignment challenging. Therefore, we performed a secondary subtyping of assemblage A by aligning our sequences with reference strains representing each sub-assemblage [4]. The detection of sub-assemblage AI was an expected finding, given its frequent association with animal hosts. To ensure the highest confidence in classification, we only assigned sub-assemblage identification to isolates that showed 100% homology to the reference strains [4]. Despite the inherent limitations in sub-assemblage classification methodologies, the *bg*-qPCR assay described herein successfully detected the prevalent *Giardia* sub-assemblages reported across both human and companion animal hosts (Table 3).

Sub-assemblages have been identified in humans, companion animals, and wildlife and are considered to have zoonotic potential [15]. Sub-assemblage BIII was predominant at the *bg* gene, whereas sub-assemblage BIV was predominant at the *gdh* gene (Table 3). Assemblage B is more polymorphic than assemblage A, and the genotyping of the three genes revealed multiple subtypes at each of the three common genotyping loci [4]. Owing to a lack of reliable discriminatory power in the *bg*, *gdh*, and *tpi* gene sequences, phylogenetic analysis is unable to distinguish between isolates. Consequently, sub-assemblages within assemblage B cannot be reliably identified using MLG [4]. Owing to the absence of *G. duodenalis* assemblage B reference sequences, sequence comparisons were using the BLASTn function only.

Although the detection of assemblage B in dogs has been infrequently documented [15], it has been reported in various studies conducted globally [55]. Several studies detected both dog-specific and zoonotic assemblages with a predominance of the zoonotic genotypes [20, 39, 51, 56–58]. The presence of assemblage A and B in dogs is likely due to cross-species transmission between humans or wildlife animals and dogs. Cats are mainly infected with cat-specific assemblage F followed by assemblage A [4]. In a study from Canada, all the eight samples typed as assemblage A and, among them, sub-assemblage AI was predominant [59]. On the basis of a meta-analysis, the prevalence of different *G. duodenalis* assemblages in cats was reported as follows: assemblage F was the most common at 55.8%, followed by assemblage A at 38.7%, assemblage D at 8.9%, assemblage C at 3.1%, assemblage E at 2.9%, and assemblage B at 2.8% [8].

Discordant results among assemblages can be explained by preferential amplification of the multilocus primers for certain assemblages or lack thereof [5, 27]. In general, however, the MLG approach is recommended for assemblage and sub-assemblage determination. In this study, the multilocus genotyping approach failed to amplify *Giardia* DNA in five samples, a finding potentially attributable to the high sensitivity of the *bg*-qPCR assay in comparison to conventional PCR. Conversely, the *bg*-qPCR assay failed to amplify DNA from three samples. We believe that the failure to amplify DNA in these three samples can be attributed to the high specificity of the qPCR locked-nucleic acid probe and potential mismatches leading to false negative results. In such situations, the probe failed to anneal owing to sequence variations, including single-nucleotide polymorphisms. Among these three samples, one sample, identified as a mixed infection of assemblages A and D by the multilocus approach, was negative by the *bg*-qPCR assay. This could also be due to the predominance of assemblage D DNA, which may have outcompeted with the amplification of assemblage A. The use of whole-genome sequencing analysis has recognized the existence of allelic sequence heterogeneity (ash) in single trophozoites and cysts of assemblage B at the genotyping loci (*gdh*, *bg*, *tpi*). The presence of ash could be due to genetic recombination, that could also be facilitated by the high occurrence of infections with mixed assemblages. This finding stresses the need for more in-depth deep sequencing of *G. duodenalis* zoonotic assemblages. 

The *bg*-qPCR assay successfully amplified potentially zoonotic *G. duodenalis* assemblages and sub-assemblages while showing no amplification of cat- and dog-specific assemblages. This is supported by the assay’s high diagnostic sensitivity and specificity, along with an almost perfect percentage of agreement with the multilocus genotyping method, which indicates that the *bg*-qPCR assay is a highly accurate tool for identifying the zoonotic *Giardia* assemblages A and B. Consequently, the *bg*-qPCR assay is recommended for ruling out the presence of potentially zoonotic strains. The high positive and negative predictive values provide valuable guidance for practitioners in the risk assessment of a *Giardia* infection in a dog or a cat.

The assemblage A- and B-specific *bg*-qPCR assays have broader applications beyond their current use. These assays could serve as a valuable tool for detecting these potentially zoonotic assemblages in fecal samples from other animal species, thereby expanding our understanding of their prevalence and distribution within a One Health framework. The assay could also be included in a multipathogen detection PCR panel to support the risk analysis of *Giardia*-infected pets as part of the clinical diagnostic workup.

## Conclusions

The *bg*-qPCR assay reliably identified the potentially zoonotic *Giardia* assemblages A and B, offering a new rapid, single-gene, qPCR-based method for assessing zoonotic risk. By reliably identifying the zoonotic assemblages A and B, which are known to infect both humans and animals, this *bg*-qPCR assay directly contributes to a One Health approach by providing a critical tool for disease surveillance and risk assessment at the human–animal interface.

## Data Availability

Data supporting the main conclusions of this study are included in the manuscript.
